# MicroRNA Expression during Bovine Oocyte Maturation and Fertilization

**DOI:** 10.3390/ijms17030396

**Published:** 2016-03-18

**Authors:** Graham C. Gilchrist, Allison Tscherner, Thomas Nalpathamkalam, Daniele Merico, Jonathan LaMarre

**Affiliations:** 1 Department of Biomedical Sciences, University of Guelph, Guelph, ON N1G 2W1, Canada; gilchrist.graham.c@gmail.com (G.C.G.); tscherner@uoguelph.ca (A.T.); 2 Program in Genetics and Genome Biology, The Centre for Applied Genomics, The Hospital for Sick Children, Toronto, ON M5G 0A4, Canada; thomas.nalpathamkalam@sickkids.ca (T.N.); daniele.merico@sickkids.ca (D.M.)

**Keywords:** miRNA, *in vitro* maturation (IVM), *in vitro* fertilization (IVF), next-generation sequencing, miR-155

## Abstract

Successful fertilization and subsequent embryo development rely on complex molecular processes starting with the development of oocyte competence through maturation. MicroRNAs (miRNAs) are small non-coding RNA molecules that function as gene regulators in many biological systems, including the oocyte and embryo. In order to further explore the roles of miRNAs in oocyte maturation, we employed small RNA sequencing as a screening tool to identify and characterize miRNA populations present in pools of bovine germinal vesicle (GV) oocytes, metaphase II (MII) oocytes, and presumptive zygotes (PZ). Each stage contained a defined miRNA population, some of which showed stable expression while others showed progressive changes between stages that were subsequently confirmed by quantitative reverse transcription polymerase chain reaction (RT-PCR). Bta-miR-155, bta-miR-222, bta-miR-21, bta-let-7d, bta-let-7i, and bta-miR-190a were among the statistically significant differentially expressed miRNAs (*p <* 0.05). To determine whether changes in specific primary miRNA (pri-miRNA) transcripts were responsible for the observed miRNA changes, we evaluated pri-miR-155, -222 and let-7d expression. Pri-miR-155 and -222 were not detected in GV oocytes but pri-miR-155 was present in MII oocytes, indicating transcription during maturation. In contrast, levels of pri-let-7d decreased during maturation, suggesting that the observed increase in let-7d expression was likely due to processing of the primary transcript. This study demonstrates that both dynamic and stable populations of miRNAs are present in bovine oocytes and zygotes and extend previous studies supporting the importance of the small RNA landscape in the maturing bovine oocyte and early embryo.

## 1. Introduction

Dynamic changes in the mRNAs and proteins expressed in oocytes prior to fertilization represent an important component of successful mammalian embryo development. Pre-ovulatory germinal vesicle (GV) oocytes remain arrested in prophase I of meiosis until a surge of gonadotropins triggers nuclear and cytoplasmic maturation events [1]. The oocyte then resumes meiosis by undergoing germinal vesicle breakdown (GVBD), extrusion of the first polar body, chromatin remodelling and cytoplasmic organelle reorganization before it is arrested again in metaphase II (MII), the point at which it becomes competent for fertilization [1,2]. Many different genes involved in cell signalling and cell cycle control must be modulated during progression through these maturation stages.

Oocyte maturation is directed in part by specific patterns of gene expression [3], even though it is generally accepted that transcription in oocytes is markedly decreased beyond the GVBD stage. Therefore, the oocyte largely relies on maternal transcripts that have been stored in the oocyte [4], or transcripts that may be delivered from the surrounding somatic cells through either gap junctions [5], or transzonal projections [6], to proceed through development. Temporal changes in the transcripts present during oocyte maturation have been recognized [7], and such changes may be due to microRNA (miRNA)-mediated regulation of transcript levels [8,9].

MicroRNAs are small non-coding RNA (ncRNA) molecules 19–24 nucleotides in length that regulate gene expression post-transcriptionally [10,11]. The canonical biogenesis pathway involves primary-microRNAs (pri-miRNAs) that are transcribed by RNA polymerase II from independent miRNA genes, although they can also be derived from introns of protein-coding genes [12,13]. Regardless of their origin, pri-miRNAs are processed into ~70 nucleotide precursor-miRNAs (pre-miRNAs) by the Drosha/DGCR8 complex, which cleaves the transcripts at the base of a stem-loop structure in the precursor [14]. Pre-miRNAs are then exported to the cytoplasm by Exportin-5 and processed by Dicer, another RNAse III enzyme, into mature miRNA duplexes [15,16]. The mature miRNA is loaded onto a protein complex containing a member of the Argonaute (AGO) family to form the miRNA-induced silencing complex (miRISC) that binds to target RNAs containing a sequence complementary to the mature miRNA. This complementary strand is then either degraded or translationally repressed [17].

MiRNAs and their associated RISC complexes normally bind to the 3’ untranslated region (3’UTR) of target mRNAs and repress protein translation or induce target mRNA decay [18]. This interaction is facilitated by the Argonaute-associated protein GW182, which functions in conjunction with other RNA binding proteins to inhibit translation or promote decay. Conversely, under circumstances such as cellular quiescence, some studies have demonstrated that miRNAs may actually enhance translation of mRNA [19,20] through mechanisms that are either directly miRNA-mediated [21], or through the indirect relief of repressors [22]. MiRNAs may be linked to transcriptional activation in a similar manner [23]. The roles and relative importance of these different mechanisms in the control of gene expression in the mammalian oocyte have not been widely characterized.

Regardless of the specific mechanisms involved and the changes induced by their presence, miRNAs are now recognized as highly effective and nearly ubiquitous regulators of gene expression, with many implications in virtually every biological process, including embryo development [24]. In embryos, studies have linked miRNAs to the degradation of maternal transcripts during the maternal-to-embryonic transition (MET) in zebrafish, murine and bovine models [25,26,27,28,29]. Recent studies have examined the transcriptome of the oocyte and specifically miRNAs using micro-array technologies and RNA sequencing (RNA-Seq) technologies. These studies have reported differences in mRNAs and miRNAs present at different stages of oocyte development. Tesfaye *et al.*, and Mondou *et al.*, [9,27] have employed heterologous micro-array approaches using human, bovine, mouse and rat miRNA probes to identify miRNAs in bovine GV and MII oocytes, as the number of registered bovine miRNAs in miRBase at the time of those studies was limited. Other studies have subsequently employed RNA sequencing strategies to identify mRNA expression patterns in bovine oocytes and embryos, and provide a list of candidate transcripts that cluster to biological functions specific to certain maturation and developmental stages. The corresponding proteome signatures in early bovine embryo development [30], provide an excellent basis for independent examination of the overall effect on the expression of predicted target genes. Collectively, these studies provide a wide-ranging perspective into the protein, transcript, and preliminary miRNA landscape in the bovine oocyte and the dynamic changes that occur during maturation. However, our present understanding is limited to a significant extent by the absence of small non-coding RNA analysis obtained by sequencing, which is both more sensitive and less subject to errors inherent in interspecies hybridization-based approaches. The purpose of the present study was to identify the population of miRNAs present during specific stages of oocyte maturation and fertilization *in vitro* through next-generation sequencing, to verify the levels of specific miRNAs by quantitative reverse transcription PCR (qRT-PCR) and to evaluate the pattern of miRNA expression in the context of the proteins present in the oocyte at those stages.

## 2. Results

### 2.1. MicroRNA Detection and Differential Expression Analysis

**Figure 1:**
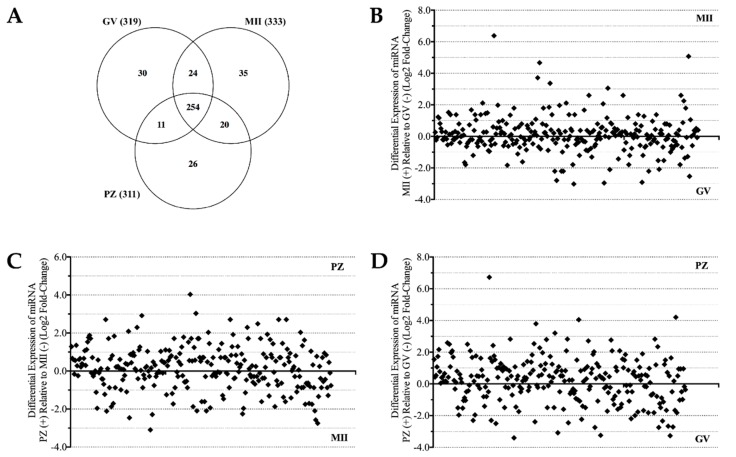
MicroRNA population distribution in germinal vesicle (GV) oocytes, metaphase II (MII) oocytes, and presumptive zygotes (PZ) as indicated by the number of unique miRNAs in each group (**A**). Differential expression analysis of miRNA populations present in: GV and MII (**B**); MII and PZ (**C**); and GV and PZ (**D**). Each represents the fold-change of individual miRNAs, exact values of each point are presented in the supplemental data.

To identify the miRNA populations in oocytes and zygotes, RNA sequence analysis was performed on small RNAs extracted from pools of (>600) GV oocytes, MII oocytes, and presumptive zygotes generating 62.4, 57.1 and 55.2 million total reads respectively. Bioinformatics processing aligned the filtered reads to known miRNA sequences (miRBase 20.0), RefSeq genes (including miRNA and ncRNA), RefSeq coding exons (excludes miRNA and ncRNA), and RFam (other ncRNA including tRNA and rRNA), and the distribution results are shown in Table 1. From the reads generated, 1.43%–1.64% mapped to annotated, mature known miRNA sequences, which represent 400 distinct miRNAs in those samples. A read frequency was assigned based on the frequency of detection of each miRNA at the specific stages evaluated. The majority of miRNAs were present in all three developmental groups, although defined sub-populations of miRNAs were present that appeared exclusively in one or two stages. (Figure 1A and Table S1).

Among the population of 254 miRNAs present at all three stages (as well as the additional 24, 20 or 11 miRNAs common between two out of three groups), differential expression analysis of the miRNA counts revealed marked changes in miRNA expression between maturation stages of oocytes/zygotes (Figure 1B–D). In total, 148–156 different miRNAs showed higher abundance with increasing maturation or after fertilization, while 109–130 different miRNAs were less abundant through these stages. A table summarizing all the specific changes in miRNA expression with their associated log_2_ fold-changes between stages is presented in the supplemental data (Tables S2–S4).

### 2.2. Quantitative Reverse Transcription Polymerase Chain Reaction (qRT-PCR) Validation of miRNAs

**Figure 2:**
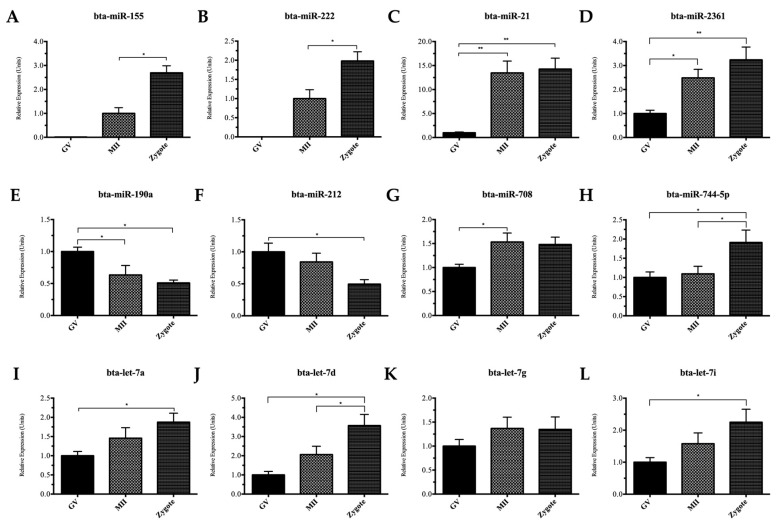
Quantitative RT-PCR (qRT-PCR) validation of mature miRNAs in GV, MII, and PZ: (**A**,**B**) expression relative to MII oocytes (1.0); (**C**–**L**) expression relative to GV oocytes (1.0). Expression was normalized to U6 and cel-miR-39-3p performed in biological triplicates of pools of 20 oocytes. Statistical significance of * indicates *p <* 0.05 and ** indicates *p <* 0.01 between specified groups.

Large numbers of oocytes and zygotes were required for sequence analysis (>600 at each stage). Due to limitations in the availability of these samples and the time and costs associated with their collection, a single pool of oocytes and zygotes from each stage was used for the sequencing portion of this study. This screening strategy effectively revealed distinct populations of miRNAs that increased, decreased or remained abundant throughout maturation, which could then be subsequently validated statistically using quantitative reverse transcription polymerase chain reaction (qRT-PCR). We selected eighteen miRNAs that were either differentially expressed between stages, or abundant throughout maturation. The data presented in Figure 2A–L, and in Figure S1A–F demonstrates that a majority of the changes in miRNAs observed the RNA sequence data were validated by qRT-PCR. Specific changes between the individual stages are presented in Tables S5–S7. Importantly, even when statistically significant changes in the “fold” expression level were not observed, the trend to higher or lower expression was preserved between the sequencing and qRT-PCR data.

### 2.3. Detection of Pri-miRNAs

**Figure 3:**
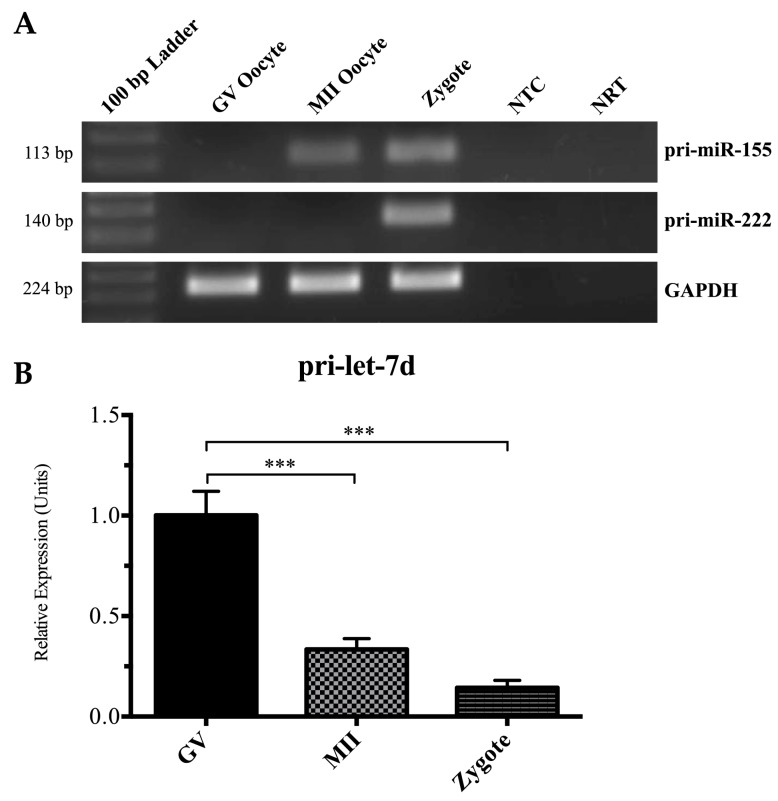
Pri-miRNA expression in oocyte maturation: pri-miR-155 and pri-miR-222 (**A**) transcripts in GV, MII and PZ with glyceraldehyde 3-phospate dehydrogenase (GAPDH) as an internal control. Detection of pri-let-7d expression using qRT-PCR in GV, MII, and PZ (**B**) expressed relative to GV oocytes using GAPDH and YWHAZ as control genes. Statistical significances (***) indicates *p <* 0.001.

To begin to characterize the molecular mechanisms behind the observed increases in expression of specific miRNAs during oocyte maturation, we designed primers to detect pri-miRNAs using RT-PCR. Increases in pri-miRNA levels should be present when transcriptional activity is increased, whereas decreases or stable expression would suggest changes in some combination of transcriptional and microprocessor activity. Levels of pri-miRNA precursors for 3 miRNAs that showed significant increases by sequencing and RT-PCR were evaluated: pri-miR-155, pri-miR-222-3p, and pri-let-7d. The results presented in Figure 3 demonstrate that pri-miR-155 and pri-miR-222-3p were both absent at the GV stage and present at the PZ stage in all pools tested (Figure 3A). Expression for these transcripts was variable at the MII stage. The levels of these pri-miRNAs were sufficiently low at one or more stages to preclude accurate quantification using qRT-PCR. In contrast to the increases seen with pri-miR-155 and -222-3p, pri-let-7d levels were highest in GV oocytes and decreased significantly upon maturation and fertilization (Figure 3B), in spite of the increase observed in the levels of mature let-7d miRNA.

### 2.4. Predicted miRNA Target Cluster Analysis

Once significant changes in the expression of specific miRNAs were validated, we next wished to identify potential targets likely to be affected by the changing oocyte miRNA landscape. In particular, we aimed to identify functional classes for the targets of key miRNAs in order to better understand potential roles of these changes in miRNA expression. To this end, we performed cluster analysis on the predicted targets of five different dynamically expressed miRNAs using TargetScan and miRDB (miR-155: 116 targets; mir-222: 140 targets; miR-21: 108 targets; let-7d: 314 targets; and miR-190a: 59 targets). The target transcript list generated by the common targets between TargetScan and miRDB was then inputted into the ClueGO application within Cytoscape to identify the probable biological interactions between the predicted target proteins as determined by the “Gene Ontology (GO) Molecular Functions” database. Functional clusters of proteins were created based on a correlative *p*-value < 0.05. The functional clusters identified represent known biological signalling pathways and other protein-to-protein interactions as determined by the Gene Ontology consortium.

**Figure 4:**
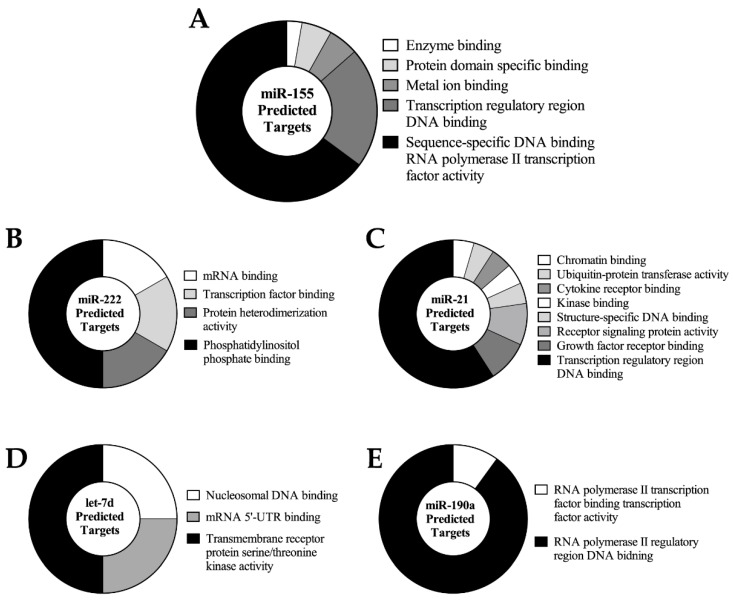
Distribution of predicted miRNA targets into functional clusters as determined by “GO: Molecular Functions” analysis with pathway significance of *p <* 0.05. The size of each functional group is proportional to the number of sub-clusters under that biological theme starting at the top (white) moving clockwise.

Upon maturation and fertilization, miR-155 levels were increased. The proteins encoded by the predicted mRNA targets of miR-155 were grouped into three functional clusters including “positive regulation of RNA metabolic process”, “negative regulation of protein metabolic process”, and “regulation of transcription from RNA polymerase II promoter” as seen in Figure 4A. Other key regulatory processes were represented in the functional groupings of the predicted targets of miR-222, -21, let-7d. miR-190a expression decreased through oocyte maturation, suggesting that the functional clusters regulated by this miRNA become more active in this period. The most consistent gene ontology clusters targeted by the miRNAs demonstrating increasing expression throughout maturation are related to transcription, protein kinase signalling, and tissue formation (Figure 4).

### 2.5. Correlation of Predicted Targets with the Bovine Oocyte and Embryo Proteome

In order to independently validate potential biological implications of miRNA changes specifically during oocyte and embryo development, we identified correlations between predicted targets of miRNAs that we had identified with proteome data from oocytes, zygotes and embryos presented in a very recent study by Deutsch *et al.* [30]. Correlations between changes in the levels of specific predicted miRNA targets present in the proteome of oocytes and zygotes, along with the percentage of identified targets that were actually increased or decreased at these stages are presented in Table 2 (a specific protein list is presented in Tables S8). MicroRNAs-155, -222, and -21 increased between the MII and PZ stages, which would normally be expected to result in a decrease in the expression of target mRNAs and/or protein. Although the putative targets themselves were not validated experimentally in this study, there is a clear trend towards consistently decreased expression of proteins encoded by mRNAs that are predicted or validated targets from two prediction software programs of miRNAs that increased between the MII and zygote stages (Table 2).

## 3. Discussion

MicroRNAs are known regulators of gene expression and protein translation that are present during oocyte and embryo development and often demonstrate rapid dynamic changes in expression [9]. The sensitivity of next-generation sequencing has allowed us to significantly extend earlier array-based studies on small RNA populations in maturating oocytes and embryos. A relatively small proportion of the small RNA reads within our data were known, annotated miRNAs (1.43%–1.64%). This corresponds well with a recent study investigating piwi-interacting RNAs (piRNA) in which it was reported that less than 1% of oocyte small RNA reads were miRNA [31]. Within our miRNA population, approximately 400 distinct miRNA sequences were detected at varying levels of expression.

In order to investigate the potential biological roles of miRNAs identified in our datasets, we cross-referenced predicted mRNA targets derived from TargetScan with the data from the oocyte and zygote proteome signature recently published by Deutsch *et al.* [30]. Of the hundreds of proteins that represent predicted targets for the 5 miRNAs chosen from our study, very few were detected in the published proteome. This small number of potential target proteins provides evidence that miRNAs present in the oocyte likely repress the basal expression and/or translation of their corresponding targets. Among the proteins that were detected in the proteome, the expression changes from MII to zygotes corresponded with high frequency to the changes expected based on our miRNA results, namely, that increasing expression of any given miRNA will decrease the expression of the protein encoded by its target and that decreasing miRNA expression will have the opposite effect. These correlations between our data the proteome characterized by Deutsch *et al.* [30] provides insight into the functional roles of miRNAs in the control of gene expression during oocyte maturation and important biological contexts for both studies, however experimental validations will enhance our understanding of miRNA guided gene regulation.

One important aspect of the present study is the function of transcripts that are repressed by the miRNAs identified here in relation to the specific developmental stages examined. It appears likely that many of the molecular processes driving oocyte maturation have the potential to be regulated by the dynamic pattern of miRNA expression. Upon germinal vesicle breakdown, it is it widely recognized that there is a widespread decrease in the level of oocyte transcriptional activity that lasts until the onset of zygotic transcription which is generally thought to happen at the 8–16 cell stage [32,33]. MicroRNA-155 is a major miRNA induced at this time point and one of the major functional clusters identified in miR-155 targets is the regulation of transcription from RNA polymerase II transcription factor activity, suggesting associations between the two processes. Other large clusters identified as targets all seem to involve transcription. This suggests that the molecular processes occurring during oocyte maturation and beyond are highly regulated by specific miRNAs.

The functional cluster analysis presented in Figure 4 is derived based on data from previously-studied gene expression data in multiple tissues [34,35] and may not apply specifically to oocyte maturation. However, in the maturing oocyte several noteworthy pathways have strong potential to be repressed by miRNAs that are expressed, including: phosphatidylinositol phosphate binding (miR-222); transcription regulatory region DNA binding (miR-21); and regulation of the protein kinase cascade activity (let-7d). Obviously further validation and investigation of these miRNAs and their targets will be necessary before definitive roles can be assigned.

Specific targets of the miRNAs identified here have been demonstrated in other cell and tissue contexts such as cancer. For example, *p53* is targeted by the let-7 family of miRNAs, and p53 is widely known to be involved in cell-cycle checkpoints [36] and to be present in the early bovine embryo [37]. Additionally, another confirmed mRNA target of miR-155 is inositol 5-phosphatase 1 (INPP5D) [38] and a decrease in INPP5D has been proven to increase AKT activity [39], a pathway involved in bovine oocyte maturation [40]. While these targets have not been proven in this study, they provide important insight into potential links for the roles these might play in oocyte maturation.

In addition to the miRNAs discussed above, it is important to note that some miRNAs were highly abundant in oocytes at all stages of maturation examined. miR-148a was the most abundant miRNA with stable expression throughout the stages examined (Figure S1). The mean number of miR-148a reads in our pools of GV, MII and PZ was 904,472, miR-10a had a mean read number of 415,282 and miR-21 had a mean of 172,010 reads in our three sequenced stages (Tables S2 and S3). The predicted targets of miR-148a cluster primarily to molecular functions involved with transcription (Figure S2). We postulate that, through repression of the many transcription factors known or predicted to be targets of this miRNA, miR-148a contributes to the low level of general transcriptional activity [4] in the maturing oocyte. Importantly, a study in *Xenopus* oocytes has demonstrated that Drosha processes pri-xtr-miR-148a more efficiently than other miRNAs [41] which may represent an additional factor contributing to the high level of this miRNA.

While the present study has extensively evaluated the miRNAs present during oocyte maturation, it is important to note that some additional aspects such as the presence of isomiRs will be important to consider in future studies. IsomiRs are variant forms of mature miRNAs that are classified based on multiple possible nucleotide variations of a canonical miRNAs arising from substitutions, shifts, and additions or deletion at either 5’ or 3’ ends [42,43]. Due to the strict algorithmic criteria required for us to confidently classify read sequences as annotated miRNAs, some proportion of our unannotated reads may represent isomiRs with functional properties that we were unable to characterize. Variants of important miRNAs may have altered targeting effects, particularly if such variation occurs at the 5’ terminus [44].

The dynamic pattern of expression of several miRNAs described in this study prompted us to investigate whether the increases in miRNA abundance observed between stages was associated with increased levels of primary miRNA (pri-miRNA) transcripts, suggesting selective increases in transcription, or with decreased levels of pri-miRNAs, suggesting increased miRNA processing. Interestingly, the expression of specific pri-miRNAs examined varied despite qRT-PCR validated increases in the levels of their cognate mature miRNAs. Expression of pri-let-7d deceased as mature let-7d levels increased, suggesting that primary transcripts were stored in the oocyte and were subject to higher levels or rates of processing throughout oocyte maturation. In contrast, pri-miR-155 and -222 were not detected in GV oocytes, but were consistently present in presumptive zygotes and variably at the MII stage using standard RT-PCR. The absence of these primary RNAs in the GV stage precluded quantitative assessment as performed for pri-let7d. The observed increase in pri-miRNA levels suggests that the primary mechanism for the increase in miR-155 and -222 expression observed through maturation is an increase in transcription of the primary miRNA and subsequent processing. This extends the work of Mondou *et al.* [27] who demonstrated transcription-dependent increases of specific miRNA precursors (miR-21 and miR-130) during oocyte maturation and early embryogenesis. Taken together, these findings suggest that pri-miRNAs are among the limited number of transcriptionally active loci in the maturing oocyte and that the cellular machinery necessary to process these transcripts becomes functional during the maturation process, however further investigation will be necessary to substantiate these findings.

Identifying a developmental stage dependent miRNA profile in bovine is important because the role of miRNA in oocyte and embryo development can vary in different species, particularly with mouse and rat models. Studies have questioned the importance of miRNAs, one study focusing in depth on let-7a specifically in murine embryo development [45,46]. However, it was discovered that mice contain an oocyte specific Dicer isoform (Dicer°) that has a preferential binding affinity to long dsRNA creating small interfering RNAs (siRNA) [47]. As a result, these siRNAs have become the predominant regulatory RNA molecule in mouse oocytes, emphasizing the importance of our results in a mammalian species other than the mouse or rat, which may also be more representative of the human embryo.

## 4. Materials and Methods

### 4.1. In Vitro Oocyte Maturation, Fertilization and Collection

Bovine (*Bos taurus*) oocyte collection and zygote production were performed as previously described [48]. Briefly, ovaries were obtained from a commercial slaughterhouse and cumulus-oocyte complexes (COCs) were collected by follicular aspiration. COCs were matured in HEPES-buffered Medium-199 (Caisson Labs, North Logan, UT, USA), supplemented with 1 µg/mL estradiol (Sigma-Aldrich, St. Louis, MO, USA), 1 µg/mL lutenizing hormone (NIH, Bethesda, USA), and 0.5 µg/mL follicle stimulating hormone (NIH). Embryos were placed in maturation media for 22 h at 38 °C in a humidified 5% CO_2_ atmosphere. Matured (MII) oocytes were fertilized with thawed bovine semen (EastGen, Guelph, ON, Canada) washed in modified TL-HEPES (Caisson labs). Approximately 1 × 10^6^ sperm were then added to each culture drop and incubated for 18 h at 38 °C in a humidified 5% CO_2_ air atmosphere. After fertilization, the remaining cumulus cells on the presumptive zygotes were manually removed by repeated pipetting in TL-HEPES. Presumptive zygotes (PZ) were then collected for analysis.

Germinal vesicle (GV) stage oocytes were collected upon COC aspiration, metaphase II oocytes (MII) were collected after 22 h of incubation in the maturation medium, and presumptive zygotes (PZ) were collected 18 h post insemination (hpi). Both GV and MII stage oocytes were treated with Hyaluronidase from *Streptomyces hyalurolyticus* (hyaluronidase) (Sigma-Aldrich), to remove adherent cumulus cells, and washed in PBS + 0.01% Polyvinyl Alcohol (PVA, Sigma-Aldrich), flash-frozen in liquid nitrogen and stored at −80 °C. Oocytes and zygotes were collected in pools ranging in size from 20 to 200, depending on the analysis. Pools of oocytes from different stages were collected from each single run, however the biological triplicates at each specific stage were always collected from IVF procedures performed on different days.

### 4.2. RNA Isolation, cDNA Synthesis, and PCR

RNA was isolated from oocytes or zygotes using the miRNeasy Micro Kit (Qiagen, Toronto, Canada) following the manufacturer’s protocol including the on column DNase digestion, and RNA was eluted in 10 mM TRIS (pH of 7.5). For qRT-PCR validation of the miRNA detected by next-generation sequencing (NGS), a cel-miR-39-3p “spike-in” control (Qiagen) was added to the QIAzol lysis step. RNA was reverse transcribed using the qScript miRNA cDNA Synthesis Kit (Quanta Biosciences, Gaithersburg, MD, USA), following the manufacturer’s protocol. Quantitative reverse transcription PCR (qRT-PCR) was carried out in a Bio-Rad CFX96™ (Bio-Rad, Missisauga, ON, Canada) instrument. The final reagent volume and concentrations in the PCR reactions were: 5 µL of PerfeCTa SYBR Green SuperMix (2×) (Quanta Biosciences), 0.2 µL of 10 µM universal primer, 0.2 µL of 10 µM miRNA specific primer that are commercially available from Quanta Biosciences, 2.6 µL Nuclease free water and 2 µL of cDNA for a final reaction volume of 10 µL. The qRT-PCR program consisted of 95 °C for 2 min, 45 cycles of 95 °C for 10 s and 60 °C for 30 s, followed by a melt curve gradient.

For pri-miRNA analysis, RNA was reverse transcribed using qScript cDNA Supermix (Quanta Biosciences) following the manufacturer’s protocol. RT-PCR reactions were: 5 µL of SsoFast EvaGreen Supermix (2×) (Bio-Rad), 1 µL of 5 µM forward and reverse primers (mixed), 2 µL Nuclease free water and 2 µL of cDNA for a final reaction volume of 10 µL. The RT-PCR program consisted of 95 °C for 2 min, 45 cycles of 95 °C for 10 s and 60 °C for 30 s. Pri-mir-155 and pri-miR-222-3p RT-PCR products were then visualized by gel electrophoresis in a 2% agarose gel, whereas pri-let-7d were qRT-PCR results were analyzed on the Bio-Rad CFX Manager Software. Pri-miR-155 forward and reverse primers were 5’-GTGGGCTGTGTGCTGTTAATG-3’, and 5’-TGGTTCCATGTGAATGCGTG-3’ respectively. Pri-miR-222-3p forward and reverse primers were 5’-ATCTAGCTGCTGGAATGTGTAG-3’, and 5’-ATCTCACTCAGGACACAGTAAC-3’ respectively. Pri-let-7d forward and reverse primers were 5’-TTTGCCCACAAGGAGGTAAC-3’ and 5’-CACCAAAGCAAGGTACCAAGG-3’ respectively. GAPDH and YWHAZ were both used as reference genes for pri-miR RT-PCR with forward and reverse sequences of 5’-TGTTGTGGATCTGACCTGCC-3’, 5’-TGTCGTACCAGGAAATGAGCTT-3’; and 5’-GCATCCCACAGACTATTTCC-3’, 5’-GCAAAGACAATGACAGACCA-3’, respectively. All qRT-PCR and RT-PCR experiments contained no-template (NTC), no-reverse transcriptase (NRT), negative and positive controls.

### 4.3. Small RNA Library Prep and Next-Generation Sequencing

RNA from a pool of 630 GV oocytes, a pool of 650 MII oocytes and a pool of 680 presumptive zygotes was extracted as previously described, and the small RNA library preparation and Illumina sequencing was performed by The Centre for Applied Genomics at The Hospital for Sick Children (Toronto, ON, Canada). Small RNA populations were prepared for sequencing using a NEBNext Multiplex Small RNA Library Prep Set for Illumina (New England BioLabs, Whitby, ON, Canada) following the manufacturer’s protocol. Briefly, 3’ adaptor primers are ligated to input RNA for 1 h at 25 °C, followed by 5’ adaptor denaturation and ligation to the RNA for 1 h at 25°C. RT reaction was performed for 1 h at 50 °C and PCR amplification was performed at 94 °C for 30 s followed by 12 cycles of 94 °C for 15 s, 62 °C for 30 s, and 70 °C for 15 s, with a final extension 70 °C for 5 min. Next-Generation Sequencing (NGS) was performed on a Illumina HiSeq 2500 system with TrueSeq v3 chemistry (Illumina, San Diego, CA, USA) using the multiplex single read protocol (50 bases).

### 4.4. Bioinformatics Analysis

Raw reads were preprocessed for adaptor trimming, quality and size selection using Trim Galore v0.2.8 (Babraham Bioinformatics, Cambridge, UK). Adapter trimming was performed with stringency 5, low-quality ends were trimmed if they were below the minimum Phred (quality score) of 20, and 17 nt (nucleotides) was the length cut-off as reads were shortened after the trimming. Reads longer than 26 nt were trimmed using the FASTX toolkit v0.6.1. The trimmed reads ranging in sized from 17–26 nt were grouped for unique sequences using scripts provided by the miRanalyzer web tool, aligned using Bowtie2 [49], and the aligned reads were classified as miRNA, RefSeq genes, RefSeq coding regions and rRNA using BEDtools v2.14.2. Of the miRNA counts in each sample, differential expression analysis was performed using the DESEq v1.10.1 Bioconductor package within R, and the raw counts were normalized and expressed as a log_2_ fold-change.

### 4.5. Statistical Analysis

For miRNA detection by quantitative RT-PCR U6 and a cel-miR-39-3p “spike-in” were used as reference genes, which both had good stability scores (>0.25) across stages. Data were expressed relative to GV samples when possible. In the cases of miR-155 and miR-222-3p, expression was determined relative to MII oocytes since target amplification in GV oocytes was low, resulting in specious enhancement of expression changes that were misleading. Comparison of means was performed on qRT-PCR data to determine statistical difference in the means using a Fisher’s LSD multiple comparisons test when comparing three means, and two means were compared using a *t*-test with Welch’s correction. All tests were conducted using Prism 6 (GradPad Software Inc., San Diego, CA, USA) and significance was determined based on a *p*-value < 0.05.

### 4.6. Predicted Target Cluster Analysis

The targets of select miRNAs of interest were determined using both TargetScanHuman software v6.2 (Whitehead Institute for Biomedical Research, Cambridge, UK) with “cow” as the selected species and miRDB software. The resulting lists from the two programs were then compared and only the predicted targets that were present on both lists were used for the functional cluster analysis. This resulting list was then analyzed in Cytoscape v3.1.1 with the ClueGO v2.1.6 plugin application [34,35]. Within ClueGO, the target lists were clustered based on the *Bos taurus* “GO Biological Processes” with a required pathway significance of *p <* 0.05. Each list of predicted miRNA targets were run separately.

## 5. Conclusions

The maturing oocyte and early embryo represent crucial stages in the development of eukaryotic organisms during which dynamic regulation of biologic processes including signalling pathways, the cell cycle, apoptosis and proliferation occurs [3,50]. The present study extends previous work by others and demonstrates the potential importance of microRNA populations in the control of gene expression that underlies these processes. Numerous relevant targets in these pathways have been characterized in other systems for the miRNAs identified here [36,51]. Our observation that the maturing oocyte transcribes and processes these important regulators further supports their importance in the developmental context. Finally, our observation that miRNA targets compared predictably with oocyte and zygote proteome signatures [30] imply an ongoing functional role in the maturing oocyte and early embryo and suggest that miRNAs have important regulatory roles in the molecular processes driving development.

## Figures and Tables

**Table 1:** Read distribution of small RNA next generation sequencing in germinal vesicle (GV) and metaphase II (MII) oocytes and presumptive zygotes (PZ).

Stage	Raw Reads (Million)	Post-Filter Reads (Million)	miRNA (%)	RefSeq Gene Reads (%)	RefSeq Coding Reads (%)	rRNA (%)	Unannotated (%)
GV	62.6	44.4	1.43	27.81	1.19	0.10	69.47
MII	57.0	39.6	1.64	27.78	1.17	0.10	69.31
PZ	55.2	33.5	1.53	27.77	1.56	0.09	69.05

**Table 2:** Correlation of predicted miRNA targets with proteins within the oocyte and zygote proteome [Deutsch *et al.*, (2014)] [30].

miRNA of Interest	MII *vs.* PZ miRNA Change	Number of Predicted miRNA Targets in Proteome	MII *vs.* PZ Common Protein Change
miR-155	Up	14	67% Down
miR-222	Up	15	56% Down
let-7d	Up	37	73% Down

## Supplementary Materials

Supplementary materials can be found at http://www.mdpi.com/1422-0067/17/3/396/s1.

## Abbreviations

The following abbreviations are used in this manuscript:

GV	germinal vesicle
GVBD	germinal vesicle breakdown
MII	metaphase II
PZ	presumptive zygote
miRNA	microRNA
ncRNA	non-coding RNA
pri-miRNA	primary-microRNA
pre-miRNA	precursor-microRNA
AGO	Argonaute
miRISC	miRNA-induced silencing complex
UTR	untranslated region
MET	maternal-to-embryonic transition
RNA-Seq	RNA sequencing
NGS	next-generation sequencing
qRT-PCR	quantitative reverse transcription PCR
GO	gene ontology
piRNA	piwi-interacting RNA
