# Supplementary material for: MicroRNA Expression during Bovine Oocyte Maturation and Fertilization
Source: Int J Mol Sci. 2016 Mar 18;17(3):396. doi: 10.3390/ijms17030396 (PMC4813251; doi:10.3390/ijms17030396)
Supplement: Supplementary file 1 [file ijms-17-00396-s001.zip › ijms-119889-publish-supplementary figures.pdf]

Supplementary Materials: MicroRNA Expression during Bovine Oocyte Maturation and Fertilization

Graham C Gilchrist, Allison Tscherner, Thomas Nalpathamkalam, Daniele Merico and Jonathan LaMarre

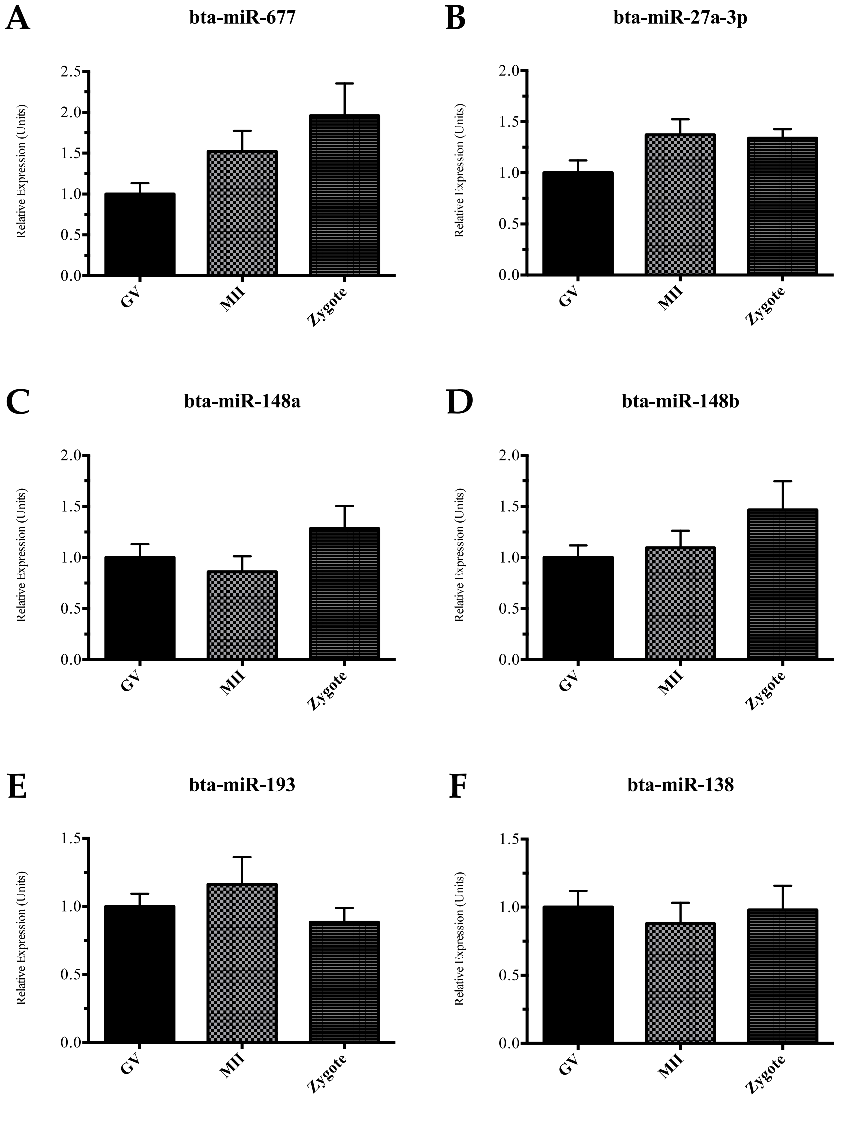

Figure S1. Quantitative PCR (qRT-PCR) validation of mature miRNAs in GV, MII, and PZ. (**A**–**F**): Expression relative to GV oocytes (1.0). Expression was normalized to U6 and cel-miR-39-3p performed in biological triplicates using pools of 20 oocytes.

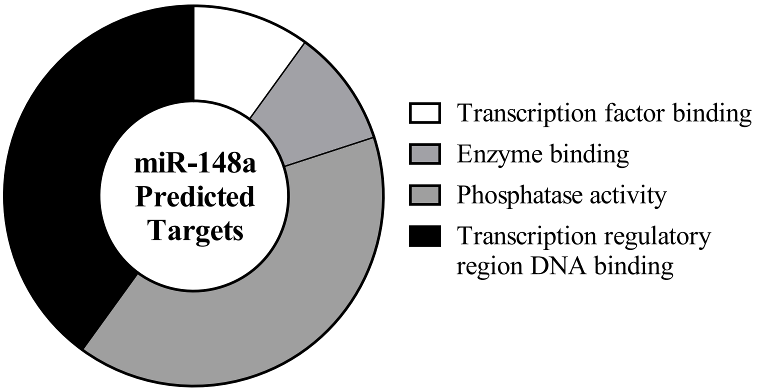

Figure S2. Distribution of predicted miR-148a targets into functional clusters as determined by "GO:Molecular Functions" analysis with pathway significance of $p < 0.05$. The size of each functional group is proportional to the number of sub-clusters under that biological theme starting at the top (white) moving clockwise.

Tables S1–S8 within Excel files in 4 different tabs.

Table S1. Presence of miRNA from Fig 1A into the respective sample in which it is detected.

Table S2. Differential expression of miRNA between GV and MII from Figure 2B.

Table S3. Differential expression of miRNA between MII and PZ from Figure 2C.

Table S4. Differential expression of miRNA between GV and PZ from Figure 2D.

Table S5. MicroRNA read count and expression fold-change from sequencing and q-RT-PCR validation results between GV and MII oocytes.

Table S6. MicroRNA read count and expression fold-change from sequencing and q-RT-PCR validation results between MII oocytes and PZ.

Table S7. MicroRNA read count and expression fold-change from sequencing and q-RT-PCR validation results between GV oocytes and PZ.

Table S8. Predicted protein targets of miRNAs present in the proteome signature from Deutsch et al. (2014) [30] with corresponding fold change between MII and Zygotes. Negative (−) values indicate higher expression in MII oocyte and positive (+) indicate the protein had a higher expression in zygotes.
